# Retinal detachment in a neonate with congenital chylothorax and purpura fulminans associated with the PAK2 genetic variant: A case report

**DOI:** 10.1016/j.ijscr.2025.111341

**Published:** 2025-04-19

**Authors:** Arijit Lodha, Majeeda Kamaluddeen, Stephanie Dotchin, Julie Lauzon, Patrick Mitchell

**Affiliations:** a Faculty of Medicine & Dentistry, University of Alberta, Edmonton, Canada; b Department of Pediatrics, Cumming School of Medicine, University of Calgary, Calgary, Canada; c Department of Ophthalmology & Visual Sciences, Cumming School of Medicine, University of Calgary, Calgary, Canada; d Department of Genetics, Cumming School of Medicine, University of Calgary, Calgary, Canada

**Keywords:** Case report, Retinal detachment, Chylothorax, Newborn, Pleural effusion

## Abstract

**Introduction and importance:**

A potential relationship between bilateral retinal detachment, chylothorax, and purpura fulminans in a female neonate with a PAK2 gene variant is not commonly reported. This emphasizes the significance of early ophthalmologic assessment in neonates with congenital chylothorax.

**Case presentation:**

A full-term female infant weighing 2.775 kg was delivered by cesarean section due to breech presentation. Prenatal imaging revealed fetal bilateral pleural effusion, suggestive of chylothorax. The neonate developed respiratory distress and purpura fulminans after birth. The absence of the red reflex in the right eye prompted a detailed ophthalmologic examination using a portable slit lamp and an indirect ophthalmoscope. The right eye revealed an ectatic pupil with posterior synechia extending from approximately 6 o'clock to 9 o'clock. Fundus examination of both eyes revealed funnel retinal detachment with multiple chronic features. A small amount of retina was draped between 4 and 7 o'clock, which may have been attached. Further ophthalmologic investigation under anesthesia, using B-scan ultrasonography and intravenous fluorescein angiography, confirmed bilateral retinal detachment. Genetic investigations revealed a *PAK2* c.1115A>T, p.(Asp372Val) variant.

**Clinical discussion:**

In addition to presenting our case report, we reviewed other recent case reports similar to ours. Retinal detachment and bilateral pleural effusions in neonates with Knobloch syndrome have been recently reported, but without purpura fulminans. Retinal detachment in neonates can result from both congenital and acquired conditions, and requires a thoughtful approach to establish the diagnosis and provide future counseling.

**Conclusion:**

Bilateral retinal detachment, chylothorax, and purpura fulminans in a neonate with a PAK2 genetic variant is uncommon. This case underscores the importance of early ophthalmologic assessment and genetic testing for both neonates and their family members.

## Highlights

• A newborn with congenital chylothorax developed purpura fulminans after effusion drainage.
• Genetic workup revealed a PAK2 variant, considered a rare occurrence.
• Ophthalmological examination revealed bilateral retinal detachment.
• Findings suggest early eye examinations and genetic testing in neonates with chylothorax.
• Early intervention may prevent visual impairment and detect potential genetic links.

## 1 Introduction

We previously demonstrated the association of congenital chylothorax in a neonate with purpura fulminans [3]. Targeted retinal detachment has not been previously reported in a neonate with chylothorax and purpura fulminans. However, two recent studies have reported the association between retinal detachment and bilateral pleural effusions in neonates with Knobloch syndrome but without purpura fulminans [1,2]. Retinal detachment in neonates can result from both congenital and acquired conditions. Managing these cases is often complex and differs considerably from standard approaches used for adults. In the pediatric population, retinal detachments account for approximately 3.2 % to 6.6 % of all cases, with an incidence ranging from 0.38 to 0.69 per 100,000 children [4]. The congenital disorders are due to congenital ocular anomalies, retinopathy of prematurity, genetic causes such as Norrie disease, familial exudative vitreoretinopathy (FEVR) [5] linked to NDP gene mutations, persistent hyperplastic primary vitreous (PHPV), congenital X-linked retinoschisis, incontinentia pigmenti, Stickler syndrome, Wagner syndrome, Knobloch syndrome [6] and idiopathic. Retinal detachment due to acquired causes are most commonly associated with trauma (44 %), followed by myopia (15 %), aphakia (10 %), and retinopathy of prematurity (ROP) (8 %), with the remaining 23 % attributed to less common factors [7,8]. The prevalence of these conditions varies by geographic region. Additionally, around 70 % of affected individuals are male, with most cases occurring between the ages of 9 and 12 years [9]. In neonates with retinal detachment, obtaining a comprehensive history is essential, with a focus on factors such as prematurity, infections, genetic syndromes, and any history of trauma. Visual acuity at presentation is generally poor, though assessing it in newborns can be challenging. In older children, vision is often significantly impaired, with an average acuity of 20/400 and the presence of nystagmus. Additional clinical findings may include esotropia, exotropia, or strabismus, which may suggest a chronic retinal detachment. Pathological changes in the contralateral eye—such as lattice degeneration, high myopia, retinopathy of prematurity, or congenital abnormalities—are seen in 89 % of cases. Bilateral retinal detachment occurs in approximately 25 % of pediatric cases. Younger children may not be able to cooperate during a detailed retinal examination in the clinic, necessitating an examination under anesthesia to ensure peripheral pathology is not overlooked. In patients with poor pupillary dilation or opacified media, a B-scan ultrasound should be performed. Considering the rarity of retinal detachment in newborns associated with congenital chylothorax, our case report is exceptionally rare and distinctive. The article has been reported in accordance with the SCARE criteria [10].

## 2 Case

This case report presents ophthalmological findings in a previously reported female infant [1], delivered at a tertiary perinatal centre at 37 weeks by cesarean section for breech presentation and maternal Hemolysis, Elevated Liver Enzymes, and Low Platelets (HELLP) syndrome. The birth weight was 2.775 kg and examination revealed mild low set ears, prominent nasal bridge and deep set eyes. The mother is a healthy gravida two, para one with no history of consanguinity or family history of lupus erythematosus or thrombosis. A detailed history from the mother indicated no history of connective tissue disorders, preeclampsia, macular degeneration, retinal tears or holes, diabetes or diabetic retinopathy, or any history of trauma, myopia and known genetic conditions in the family. Prenatal imaging at 20 weeks revealed bilateral pleural effusion, predominantly on the left side, suggestive of chylothorax. The etiology of chylothorax was unknown in the presence of a patent and intact thoracic duct. Respiratory distress improved with mask intermittent positive pressure ventilation.

A series of medical interventions followed, including thoracocentesis to drain the chylous fluid. This was followed by the onset of a generalized, well-demarcated erythematous cutaneous macules rapidly progressing to blue-black purpuric lesions. Purpura fulminans was associated with transient low platelet levels, potentially attributed to Disseminated Intravascular Coagulation (DIC), which resolved with fresh frozen plasma infusions. Specialty consultations were initiated, including haematology, genetics, dermatology, and infectious diseases.

Genetic investigations including whole exome sequencing, did not report any definite causative variants; however, it did reveal a de novo variant of unknown clinical significance in the PAK2 gene, *PAK2* c.1115A>T, p.(Asp372Val). In the first few months of life, the child displayed uncoordinated eye movements, absence of the red reflex in the right eye, poor focus, difficulty recognizing faces, trouble with depth perception and distinguishing the relative distance of objects, as well as a lack of eye-body coordination. This prompted an ophthalmology consult and examination using a portable slit lamp and indirect ophthalmoscope.

The slit-lamp examination of the right eye revealed an ectatic pupil, with posterior synechia extending from approximately 6 o'clock to 9 o'clock position. A membrane was also noted on the anterior surface of the iris inferiorly. The patient's natural lens was well centered and clear, but due to the pupil's distortion, zonules were visible superonasally. Slit lamp examination of the left eye was normal. Fundus examination of the right eye revealed a funnel retinal detachment with multiple chronic features. A careful examination revealed the presence of no calcium crystals. Fundus examination of the left eye also revealed a retinal detachment, this time in a knife-fold configuration emanating from the optic nerve head to the inferotemporal periphery. A small amount of retina was draped between 4 o'clock and 7 o'clock and may have been attached. Intraocular pressure measured by Tono-Pen was 10 mmHg in both eyes.

**Fig. 1:**
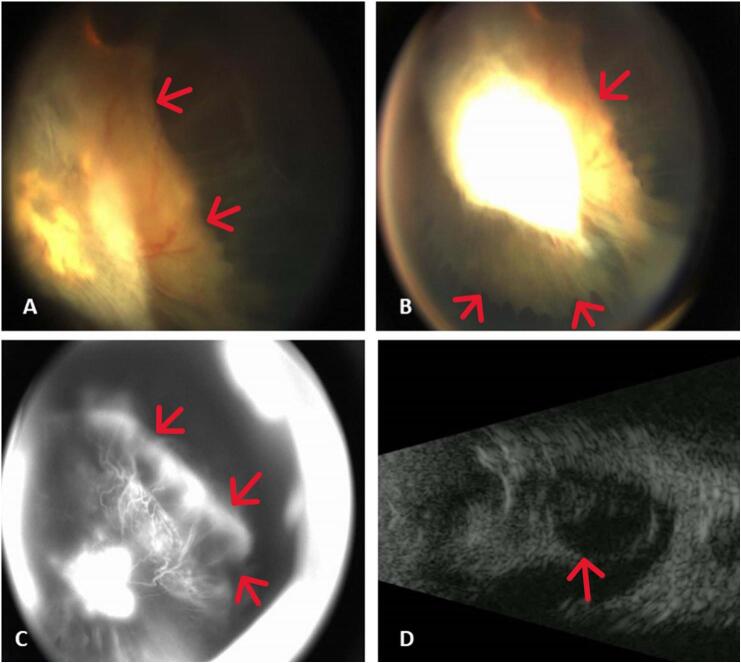
(Right eye). A. Retcam color fundus photo, focused posterior. B. Retcam color fundus photo, focused anterior showing ciliary body traction and funnel retinal detachment. C. Fluorescein angiography, late image. D. B-Scan ultrasound confirming total funnel retinal detachment.

**Fig. 2:**
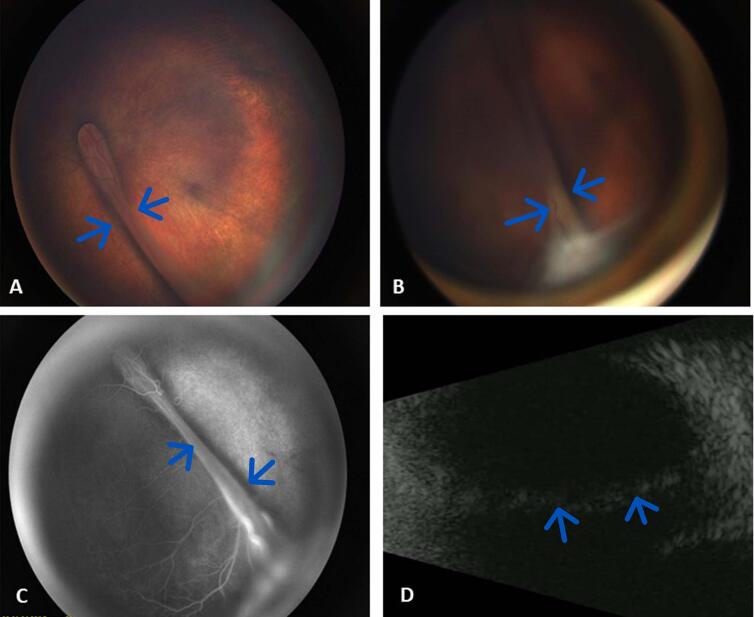
(Left eye). A. Retcam color fundus photo, focused posterior. B. Retcam color fundus photo, focused anterior showing knife fold retinal detachment with anterior fibrosis to posterior lens capsule. C. Fluorescein angiography, late image. D. B-Scan ultrasound confirming knife fold configuration retinal detachment emanating from the optic nerve and propagating towards the inferotemporal periphery.

The ophthalmological findings of bilateral retinal detachment were subsequently confirmed with an examination under anesthesia, using B-scan ultrasonography (Fig. 1) and intravenous fluorescein angiography (Fig. 2). Based on the clinical appearance, the consulting ophthalmologist's clinical impression was severe familial exudative vitreoretinopathy [5].

Given the severity of the retinal findings, limited surgical options, high risk of complications, and uncertain prognosis due to underlying genetic conditions following discussion of the potential risks and benefits of surgery with the patient's parents, the decision was made to forgo surgery and take a conservative approach.

The patient's eyes were re-examined under anesthesia at 21 months of age. Intraocular pressures, measured immediately after the induction of general anesthesia with tetracaine instilled, were 10 mmHg in the right eye (OD) and 19 mmHg in the left eye (OS) via tonopen.

Anterior segment examination using a portable slit lamp and indirect ophthalmoscope revealed the following in the right eye: normal conjunctiva, clear cornea, slightly narrowed and quiet anterior chamber, corectopia of the superonasal iris, and a cataract. In the left eye, the findings included normal conjunctiva, clear cornea, a deep and quiet anterior chamber, normal iris, and clear lens. During the detailed fundus examination, the ophthalmologist was unable to obtain a clear view; however, the left fundus showed a normal optic nerve but a folded retina, normal posterior vessels with anterior nasal nonperfusion and no neovascularization, as well as a temporal anterior retinal fold. The B-scan of the right eye showed a closed funnel. The fluorescein angiogram of the left eye demonstrated normal vessel filling in zone 1, with nasal nonperfusion, no leakage, and minimal filling of the superotemporal retina. The procedure, performed at 21 months of age, was completed without any complications.

## 3 Discussion

As far as we are aware, this represents an uncommon case of bilateral retinal detachment in a neonate with congenital chylothorax and purpura fulminans, associated with a PAK2 genetic variant.

The PAK2 gene encodes a protein kinase from the p21-activated kinase (PAK) family. PAK proteins regulate various processes that involve dynamic cytoskeletal reorganization, such as cell adhesion, migration, cell progression, apoptosis, and proliferation [2].

Knobloch syndrome is a rare genetic disorder that primarily affects the eyes, and in some cases, the central nervous system and lungs. It is marked by various ophthalmological issues, including retinal detachment, which often leads to vision impairment, vitreoretinal degeneration, macular abnormalities, corectopia, and cataracts. There are two types of Knobloch syndrome: Type 1, caused by mutations in the COL18A1 gene, which disrupts collagen production, a key structural protein; and Type 2, linked to mutations in the PAK2 gene, which regulates cell growth and signaling. Besides eye-related issues, individuals with Knobloch syndrome may also have craniofacial abnormalities, such as a prominent nasal bridge, deep-set eyes, or low-set ears, along with neurological problems like hydrocephalus or developmental delays. Knobloch type 1 is inherited in an autosomal recessive pattern, while Knobloch type 2 has been associated with heterozygous variants in the kinase domain of PAK2. Early diagnosis and management, especially ophthalmological evaluation, are vital for improving outcomes.

Domenach et al. describes a heterozygous variant in PAK2 in which a fetus diagnosed with severe bilateral pleural effusion at 26 + 6 weeks' gestation. Post-drainage, partial resolution of the effusion was noted. Analysis of the pleural fluid revealed a predominance of lymphocytes and a few macrophages, suggestive of chylothorax, though triglyceride levels were not elevated. Prenatal trio exome sequencing identified a novel de novo *PAK2* missense variant, NM_002577.4:c.836A>C, p.(Gln279Pro), classified as likely pathogenic. No other variants were detected in genes known to be associated with intrauterine pleural effusions. Interestingly, a hallmark of Knobloch syndrome is the consistent presence of ophthalmic defects—an aspect notably absent in this fetus. Instead, the clinical course was marked by bilateral pulmonary hyperplasia, pulmonary hypertension, and lactic acidosis. Seven cases of PAK2-associated Knobloch syndrome, including our case, were reviewed [1,3,6,11,12]. Many of the typical features of Knobloch syndrome were observed, including ophthalmologic, pulmonary, and cardiac anomalies [1,3,6,11,12]. All of these PAK2 related Knobloch cases had de novo variants in the kinase domain [1,3,6,11,12]. A literature search identified two prior cases of bilateral retinal detachment associated with chylothorax in Knobloch syndrome type 2 [1,2] but without purpura fulminans. Based on our case report and two other cases, we recognize that the combination of pleural effusions and retinal detachment in the presence of PAK2 genetic variant may suggest the diagnosis of Knobloch syndrome type 2.

However, the ocular changes in our patient, such as retinal detachment, may have several other potential explanations. These changes may be associated with severe vitreoretinal degeneration, retinal detachment, and macular abnormalities [1]. Other potential mechanisms include thrombosis related to purpura fulminans, or may suggest conditions such as Familial Exudative Vitreoretinopathy (FEVR), a severe form of persistent fetal vasculature, or the rare Stickler syndrome.

In FEVR, the peripheral retina is primarily affected, leading to areas of non-perfusion and abnormal blood vessel growth, which can increase the risk of retinal traction and detachment. This can also result in fibrosis and scar tissue formation, pulling the retina away from its underlying layers, which is more common in severe cases of FEVR. Retinal detachment present at birth may involve peripheral retinal non-perfusion, vitreous hemorrhage, and vascular abnormalities. The visual prognosis for neonates with retinal detachment due to FEVR depends on several factors, including early detection and intervention, the extent and progression of the detachment, the presence of complications like vitreous hemorrhage or retinal scarring, and the age at presentation. The younger the child, the more challenging it is to achieve good visual outcomes, particularly if the detachment is extensive. Management options for FEVR include conservative observation, laser photocoagulation, vitrectomy with scleral buckling [9], intravitreal anti-VEGF injections, as well as genetic counseling and family screening. Vitrectomy with release of posterior traction is crucial in younger patients with vascularly active fibrovascular proliferation, while scleral buckling may be necessary for cases with peripheral traction located anterior to the equator. In most instances, peripheral thermal treatment should be applied to all ischemic areas to help reduce peripheral neovascularization [13]. In older children, favorable outcomes can be achieved, although multiple surgical interventions may be required [14]. In our case, Stickler syndrome was excluded as the neonate did not exhibit any craniofacial dysmorphic features, such as cleft palate, a small lower jaw, tongue displacement, or a flat facial appearance. Additionally, the neonate had no sensorineural or conductive hearing impairments, no skeletal or joint abnormalities, and no cataracts. FEVR remains a possibility, as it is marked by abnormal retinal angiogenesis that results in incomplete peripheral retinal vascularization, potentially leading to retinal ischemia and detachment.

We approached the family for additional genetic investigations as the exome analysis was performed as the ophthalmologic issues were still evolving, but they declined to proceed with any further testing. It is therefore possible that the patient's clinical features may be due in part by another underlying genetic variant however, recent evidence from the literature suggests that this PAK2 variant is contributory to our patient's clinical features.

## 4 Conclusion

This rare case report proposes a probable link between bilateral retinal detachment, chylothorax, and purpura fulminans in a neonate with a PAK2 genetic variant. It underscores the importance of early ophthalmologic assessment and genetic testing for both neonates and their family members diagnosed with congenital chylothorax, to promptly identify retinal detachment and facilitate timely interventions to reduce the risk of vision impairment. In our perspective, the establishment of a national-level registry encompassing neonates afflicted with congenital chylothorax would be a worthy endeavour. By amalgamating information on this condition with its associations, we envision a comprehensive resource that not only aids in refining clinical approaches, but also fosters advancement in our understanding of Knobloch syndrome and potentially associated conditions.

## Patient consent

Consent to publish the case report was obtained. This report does not contain any personal information that could result in patient identification.

## Ethical approval

This study did not require ethical approval as it is a case report. However, informed consent has been obtained and can be provided on request.

## Guarantor

Majeeda Kamaluddeen

## Funding

None.

## Author contribution

**Arijit Lodha:** Writing original draft.

**Majeeda Kamaluddeen:** Provided clinical care, conceptualization, data acquisition, and review of the draft.

**Stephanie Anne Dotchin:** Clinical care, review and editing, data curation.

**Julie Lauzon:** Review, editing, and providing genetic opinion.

**Patrick Mitchell:** Clinical care, review, editing, and providing images.

## Conflict of interest statement

The authors declare that they have no known competing financial interests or personal relationships that could have appeared to influence the work reported in this paper.

## References

[1] Schnur R.E., Dvoracek L., Kalsner L. (Oct 2024). New kinase-deficient PAK2 variants associated with Knobloch syndrome type 2. Clin. Genet..

[2] Domenach L., Rooryck C., Legendre M., Bouchghoul H., Beneteau C., Margot H. (Feb 24, 2025). Antenatal phenotype associated with PAK2 pathogenic variants: bilateral pleural effusion as a warning sign. BMC Med. Genomics.

[3] Joseph C.J., Lodha A., Thomas S.R. (2023). Case report: blotchy skin in a puffy neonate: is there a new association?. Front. Pediatr..

[4] Meier P. (Sep 2008). Retinal detachment in children: differential diagnosis and current therapy. Klin Monbl Augenheilkd..

[5] Gilmour D.F. (Jan 2015). Familial exudative vitreoretinopathy and related retinopathies. Eye (Lond.).

[6] Antonarakis S.E., Holoubek A., Rapti M. (Dec 17, 2021). Dominant monoallelic variant in the PAK2 gene causes Knobloch syndrome type 2. Hum. Mol. Genet..

[7] Winslow R.L., Tasman W. (Jun 1978). Juvenile rhegmatogenous retinal detachment. Ophthalmology.

[8] Gan N.Y., Lam W.C. (Oct–Dec 2018). Retinal detachments in the pediatric population. Taiwan J. Ophthalmol..

[9] Nuzzi R., Lavia C., Spinetta R. (2017). Paediatric retinal detachment: a review. Int. J. Ophthalmol..

[10] Sohrabi C., Mathew G., Maria N. (2023). The SCARE 2023 guideline: updating consensus Surgical CAse REport (SCARE) guidelines. Int. J. Surg..

[11] Werren E.A., Kalsner L., Ewald J. (Apr 22, 2024).

[12] Wilson C., Aftimos S., Pereira A., McKay R. (Jul 7, 1998). Report of two sibs with Knobloch syndrome (encephalocoele and viteroretinal degeneration) and other anomalies. Am. J. Med. Genet..

[13] Yamane T., Yokoi T., Nakayama Y., Nishina S., Azuma N. (Nov 2014). Surgical outcomes of progressive tractional retinal detachment associated with familial exudative vitreoretinopathy. Am. J. Ophthalmol..

[14] Sen P., Singh N., Rishi E. (Jun 2020). Outcomes of surgery in eyes with familial exudative vitreoretinopathyassociated retinal detachment. Can. J. Ophthalmol..

